# Mesoscale Polarization by Geometric Frustration in Columnar Supramolecular Crystals

**DOI:** 10.1002/anie.201612122

**Published:** 2017-03-20

**Authors:** Christoph S. Zehe, Joshua A. Hill, Nicholas P. Funnell, Klaus Kreger, Kasper P. van der Zwan, Andrew L. Goodwin, Hans‐Werner Schmidt, Jürgen Senker

**Affiliations:** ^1^ Inorganic Chemistry III University of Bayreuth Universitätsstrasse 30 95447 Bayreuth Germany; ^2^ Department of Chemistry University of Oxford Inorganic Chemistry Laboratory South Parks Road Oxford OX1 3QR UK; ^3^ Current address: ISIS Rutherford Appleton Laboratory Chilton Didcot OX11 0QX UK; ^4^ Macromolecular Chemistry I University of Bayreuth Universitätsstrasse 30 95447 Bayreuth Germany

**Keywords:** Ising model, organic ferroelectrics, self-assembly, supramolecular chemistry, total X-ray scattering

## Abstract

Columnar supramolecular phases with polarization along the columnar axis have potential for the development of ultrahigh‐density memories as every single column might function as a memory element. By investigating structure and disorder for four columnar benzene‐1,3,5‐trisamides by total X‐ray scattering and DFT calculations, we demonstrate that the column orientation, and thus the columnar dipole moment, is receptive to geometric frustration if the columns aggregate in a hexagonal rod packing. The frustration suppresses conventional antiferroelectric order and heightens the sensitivity towards collective intercolumnar packing effects. The latter finding allows for the building up of mesoscale domains with spontaneous polarization. Our results suggest how the complex interplay between steric and electrostatic interactions is influenced by a straightforward chemical design of the molecular synthons to create spontaneous polarization and to adjust mesoscale domain size.

Organic materials featuring ferroelectric polarization^1^ are attractive candidates for easily processable and low‐cost electric sensors,^2^ electro‐optics,^3^ as well as non‐volatile memory devices.^4^ The fundamental requirement for these materials is spontaneous and switchable polarization. In supramolecular solids^1^, ^3^ and liquid crystalline (LC) phases,^5^ the latter is generally induced by ordering either permanent molecular dipoles or supramolecular dipole moments that are generated or enhanced by the assembly.^6^ In particular, materials with polarization along the columnar axis (referred to as axially polar) have gained increasing interest owing to their potential applicability for ultrahigh‐density memories, where individual columns might ultimately act as memory elements.^7^, ^8^

Recently, the first example of an intrinsically ferroelectric, axially polar LC with a remnant polarization of 1.7 μC cm^−2^ was obtained by columnar stacking of a phthalonitrile derivative.^9^ Kemerink, Sijbesma, and co‐workers reported a similar polarization of up to 2 μC cm^−2^ for thin films of oriented LCs^10^ that consisted of benzene‐1,3,5‐trisamides (BTAs)^11^ with long aliphatic side groups. Although the polarization could be induced and switched by electric fields, its stabilization was only possible by freezing the LC state.^10^, ^11^ Therefore, one of the biggest challenges for axially polar materials remains the creation and control of spontaneous polarization in the absence of external stimuli. The latter requires the counterbalance of the electrostatic interactions between the dipoles of neighboring columns in a side‐by‐side arrangement, which inevitably favors an *anti* alignment of the columnar polarization, resulting in non‐polar phases.^3^ Hence, suitable columnar materials with spontaneous and stable polarization are rare up to now, and for the few observed cases,^8^ its emergence remained unexplained.^7^, ^8^, ^9^, ^12^, ^13^, ^14^

Herein, we present a structural study of four purposely synthesized BTAs with the aim of investigating the origin of spontaneous polarization for axially polar systems in their solid state, which offers the unique possibility to study dipole order governed solely by intrinsic interactions. In general, BTAs are based on a benzene core that is linked to peripheral groups via three amide bonds in the 1‐, 3‐, and 5‐positions (Figure 1 a). The formation of threefold intermolecular hydrogen bonds in a helical arrangement drives the molecular self‐assembly into well‐ordered columns (Figure 1 b, left), in which all carbonyl bonds are aligned in the same direction along the columnar axis. The individual dipole moments of these bonds add up to macrodipoles^10^ along the columnar axis (Figure 1 b, left). In a side‐by‐side arrangement of two columns, the electrostatic interactions prefer an *anti* alignment of the macrodipoles (Figure 1 c). Simultaneously, the peripheral groups give rise to a corrugated surface topography for the supramolecular aggregates (Figure 1 b, right), imposing steric restraints (Figure 1 c) that may favor parallel or antiparallel arrangements depending on subtle structural details of the peripheral groups. As van der Waals forces favor dense packings of neighboring columns, hexagonal rod packings^14^ are often induced (Figure 1 d). In such packings, however, the macrodipole interaction between neighboring columns becomes frustrated as it is not possible to simultaneously align all dipoles antiparallel relative to their nearest neighbors (Figure 1 d). This frustration reduces the contribution of the macrodipole interactions to the lattice energy and thus amplifies the influence of steric restraints. If the latter favors the parallel alignment of neighboring columns, stable ferroelectric domains should be feasible for axially polar materials.

**Figure 1:**
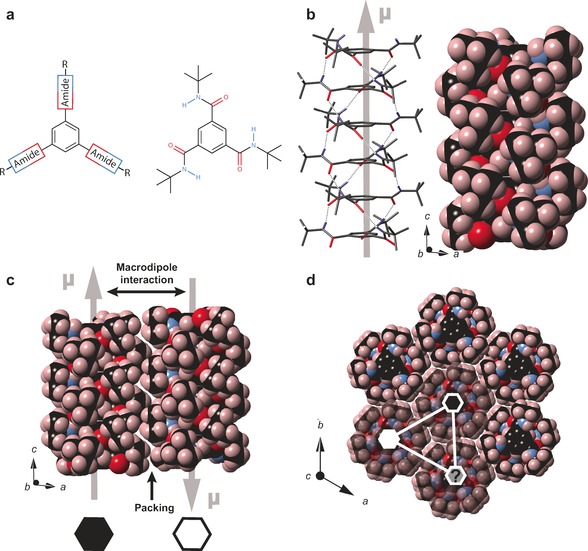
a) Basic design scheme of BTAs, which consist of a benzene core linked to peripheral groups R (such as *tert*‐butyl moieties) via amide bonds in the 1‐, 3‐, and 5‐positions. b) Side view of a columnar stack of six BTA molecules (left); the hydrogen bonds are indicated as dotted lines (all non‐NH protons omitted for clarity). The macrodipole is highlighted as a gray arrow. A space‐filling model of the same stack is depicted on the right. c) Side view of two columns in antiparallel orientation, where the direction of the macrodipole is symbolized by black and white hexagons. d) Top view of an ensemble of seven stacks in hexagonal rod packing indicating possible geometric frustration.

Based on this model, compounds **1**–**4** (Figure 2 a) were selected, for which we expected a varying ratio of macrodipole and steric interactions. Single crystals were grown either by solvent evaporation or by sublimation and analyzed by single‐crystal X‐ray diffraction (for crystallographic details, see the Supporting Information, Section S2). All four BTAs crystallize in hexagonal rod packings (Figure 2 b), which is in agreement with previous studies on **1** 15 and **2**.^16^ Whereas **1**, **2**, and **4** exhibit similar intercolumnar distances *d*_cc_ of 14.1 Å, 14.5 Å, and 13.9 Å, the longer peripheral groups of **3** increase the *d*_cc_ value to 17.1 Å. The C=O centered linkages of **1** and **3** feature higher torsional flexibility of the amide groups with mean side‐chain torsions of *Φ*(C_ar_C_ar_C_O_O)≈39.5° (**1**) and 34.6° (**3**) compared to the N‐centered systems with *Φ*(C_ar_C_ar_C_O_O)≈35.1° (**2**) and 23.7° (**4**). Larger torsion angles cause a steeper inclination of the C=O bonds towards the columnar axis and will thus lead to larger macrodipoles.

**Figure 2:**
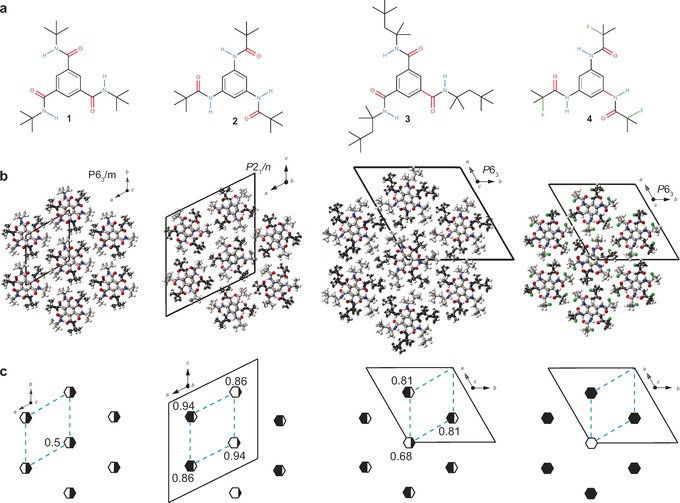
a) BTA molecules **1**–**4**. b) Bragg structure solutions viewed along the direction of the molecular stacks, coinciding with the direction of the polar axes of the indicated space groups. c) Schematic representation of the probabilities for up‐ and down‐oriented columns within one stack (major components displayed), which are additionally symbolized by the amount of black and white in each hexagon. The dashed blue lines indicate the unit cells of the underlying hexagonal or pseudo‐hexagonal (in the case of **2**) lattices whereas the black solid lines demark the unit cells found by single‐crystal X‐ray diffraction.

To derive a more quantitative picture of the macrodipole strengths, we estimated the average molecular dipole moments *p* representative for the columnar dipole for the smaller molecules **1**, **2**, and **4** by quantum‐chemical calculations on finite clusters taken from the single‐crystal structure solutions (Section S3). In line with the structural considerations, *p* amounts to 12 D and 11 D for **1** and **2**, respectively, and to 6.5 D for **4**. For the latter, the *p* value is reduced further because of the *anti* alignment of the C−F and C=O bonds, which is induced by intramolecular NH⋅⋅⋅F hydrogen bonds (Figure 2 a, b and Figure S4 b).^17^ In the case of **3**, we expect a value close to the one derived for **2** as their mean torsion angles are similar. As a consequence, the strongest macrodipole interactions arise for **1** (*p=*12 D, *d*_cc_=14.1 Å), closely followed by **2** (11 D, 14.5 Å) and a significant reduction for **3** (11 D, 17.1 Å) owing to the larger *d*_cc_ value and **4** (6.5 D, 13.9 Å) based on the smaller *p* value.

Remarkably, for **1**,^15^ **2**, and **3**, each stack appears as a superposition of two columns with opposite macrodipole orientations in the conventional structure solution (Figure 2 c). As Bragg diffraction arises from spatially averaged electron density within the coherence length of the X‐ray beam (ca. 100 nm), this reflects disorder of the macrodipole orientations in individual unit cells.^18^ For **1**, both orientations are equally present (Figure 2 c), and hence on average, each stack has a 50 % probability to be either in the up or the down state. In contrast, we observed superstructures with a stripe‐type macrodipole arrangement for **2** and with a honeycomb pattern for **3** and **4** (Figure 2 c). For the latter BTAs, in each stack, one particular column orientation is more likely than the other as indicated by the color code presented in Figure 2 c. Nevertheless, the unit cells of **1** and **2** contain no net macrodipole moment as equal amounts of up‐ and down‐oriented columns are present. For **3** and **4**, the unit cells contain an excess of one orientation, which leads to a net polarization along the direction of the columnar axis.

In addition to the sharp Bragg reflections, selected layers of reciprocal space exhibit intense, structured, diffuse scattering for compounds **1**, **2**, and **3** (Figure 3 a).^18^ In all cases, the diffuse intensities are confined to layers perpendicular to the stacking direction (Figure S2). The diffuse scattering explains the disorder for up‐ and down‐oriented columns as described above (Figure 2 c) and suggests deviations from the average structures for neighboring columns within a coherence length below 100 nm. In contrast, the columns are rather well‐ordered along the stacking direction on significantly larger length scales. To develop models that reproduce the observed diffuse scattering pattern, we found it to be sufficient to position supramolecular columns in a hexagonal rod packing, with only the up and down orientation of the macrodipoles subject to disorder, while the packing along the stacking direction is ordered over the whole column. We then predicted the ferroelectric and antiferroelectric alignments of neighboring macrodipoles based on a 2D Ising model.^19^ Here, the energy of the system is defined based on two effective coupling constants $J_{1}$ and $J_{2}$ , which describe the sum of both the electrostatic and steric interactions between nearest neighbor (n.n.) and next‐nearest neighbor (n.n.n.) columns.^19^ $J_{1}$ and $J_{2}$ are subsequently varied until the best match between the simulated and experimental diffraction patterns (Figure 3 a, b) is reached (for a description of the technical procedure, see Section S1.4).

**Figure 3:**
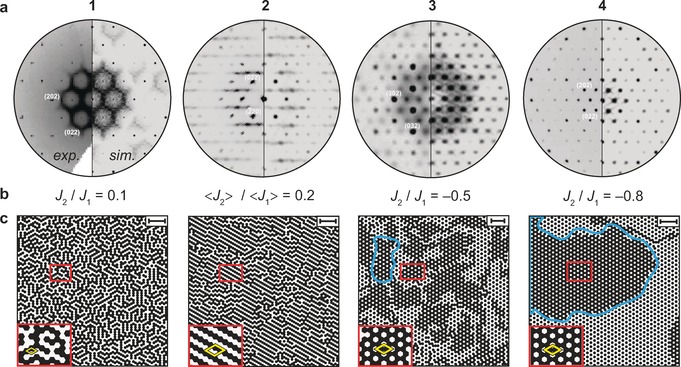
a) The (*hk*2) planes for **1**, **3**, and **4** and the (*h*2*k*) plane for **2** of reconstruction of the reciprocal space, together with simulations (experimental picture of **1** taken from Ref. 15). These planes are perpendicular to the stacking direction. b) Ratios for the refined coupling constants $J_{1}$ and $J_{2}$ of the Ising model simulations for **1**–**4**. Owing to the reduced crystallographic symmetry in the case of **2**, average values are given (Figure S1). All constants were normalized according to $J_{1}/kT=1$ , with *T* being the simulation temperature. c) Resulting arrangement of up‐ and down‐oriented macrodipoles over approximately 70×70 columns. The red boxes contain magnifications of local features, and the crystallographic unit cells are indicated in yellow (Figure 2 b). The blue lines emphasize domains exhibiting net polarization for **3** and **4**. The scale bars in the top right corners correspond to a distance of 10 nm.

The resulting arrangements of up‐ and down‐oriented macrodipoles (Figure 3 c) reproduce the diffuse scattering pattern (Figure 3 a) with almost perfect agreement. **1** and **2** both exhibit disorder on a local scale with preferential stripe‐type antiferroelectric arrangements between neighboring columns. In contrast, for **3** and **4**, mesoscale domains with a honeycomb structure carrying spontaneous polarization are formed. While the average domain size is on the order of 20–30 nm for **3**, the domains become significantly larger (50–70 nm) for **4**. Furthermore, the excellent match between the observed and simulated X‐ray powder diffraction data (Figures S5–S8) demonstrates that both the disorder and the mesoscale domain formation are inherent properties of the bulk materials. Although domains with spontaneous polarization have been suggested for axially polar phases before,^7^, ^8^, ^9^, ^12^, ^20^ our data provide the first experimental evidence for their existence. For such small domains, other common methods, such as the second harmonic generation effect, are inconclusive^21^ or change the domain structure as for pyroelectric measurements. In our case, total scattering proves to be the method of choice for probing small domains without affecting the spontaneous polarization.

For the two compounds with the larger macrodipole interactions (**1** and **2**), both $J_{1}$ and $J_{2}$ are positive, indicating that the electrostatic interaction, and thus an antiferroelectric alignment between n.n. and n.n.n. columns, dominates. This inevitably leads to disorder for up‐ and down‐oriented columns on local length scales and non‐polar unit cells (Figure 2 c and Figure 3 c). In contrast, for **3** and **4**, where the electrostatic interaction is significantly reduced, $J_{1}$ and $J_{2}$ bear opposite signs with $J_{1}>0$ and $J_{2}<0$ . Consequently, only n.n. columns tend to be *anti*‐aligned while n.n.n. stacks favor a parallel alignment. The latter occurs only when the steric interactions and collective packing effects^22^ become stronger than the electrostatic forces. The tendency for an opposite alignment for n.n. and n.n.n. columns in turn is the origin of the formation of mesoscale domains with spontaneous polarization and polar unit cells (Figure 2 c and Figure 3 c). Based on the calculated molecular dipole moments, the polarization within these domains reaches values of 1.6 μC cm^−2^ and 1.4 μC cm^−2^ for **3** and **4**, which are among the largest values obtained for axially polar phases to date.^9^, ^10^, ^11^, ^12^, ^13^, ^23^

Established approaches for creating ferroelectric order make use of supramolecular interactions between individual molecules to counteract the electrostatic interaction.^3^ In contrast, we have shown that for axially polar phases, polarization emerges from weak steric interactions between self‐assembled supramolecular columns if the systems are prone to geometric frustration. As these interactions are encoded in the molecular structure, the latter drive the dipole ordering and the length scale of domain formation. As such, it is possible to move between nonpolar stripe‐type and polar honeycomb phases in a 2D Ising phase diagram (Figure 4).^19^ Our finding illustrates emerging complexity^24^ and is an intriguing example of introducing hierarchical order in supramolecular systems.^25^ With small, but systematic chemical modifications of the molecular synthons, a broad range of antiferroelectric and ferroelectric domain structures are accessible. Counterintuitively, mesoscale domains are maximized for systems where dipolar interactions are reduced. Following this idea for other columnar materials may lead to the development of a wide variety of solid and liquid‐crystalline, axially polar columnar ferroelectric materials.

**Figure 4:**
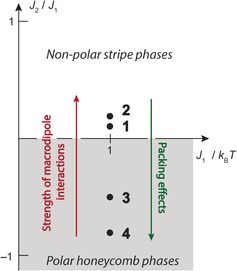
The ground‐state phase diagram^19^ of the simple 2D Ising model exhibiting antiferroelectric nearest ($J_{1}>0$ ) and varying next‐nearest neighbor interactions.

## Conflict of interest

The authors declare no conflict of interest.

## Supporting information

As a service to our authors and readers, this journal provides supporting information supplied by the authors. Such materials are peer reviewed and may be re‐organized for online delivery, but are not copy‐edited or typeset. Technical support issues arising from supporting information (other than missing files) should be addressed to the authors.

Supplementary

## References

[1] S. Horiuchi , Y. Tokura , Nat. Mater. 2008, 7, 357–366. 18432209 10.1038/nmat2137

[2] J. F. Scott , Science 2007, 315, 954–959. 17303745 10.1126/science.1129564

[3] A. S. Tayi , A. Kaeser , M. Matsumoto , T. Aida , S. I. Stupp , Nat. Chem. 2015, 7, 281–294. 25803466 10.1038/nchem.2206

[4] K. Asadi , D. M. de Leeuw , B. de Boer , P. W. M. Blom , Nat. Mater. 2008, 7, 547–550. 18552851 10.1038/nmat2207

[5] S. T. Lagerwall , Ferroelectrics 2004, 301, 15–45.

[6a] S. Horiuchi , F. Ishii , R. Kumai , Y. Okimoto , H. Tachibana , N. Nagaosa , Y. Tokura , Nat. Mater. 2005, 4, 163–166; 15665837 10.1038/nmat1298

[6b] D.-W. Fu et al., Science 2013, 339, 425–428; 23349285 10.1126/science.1229675

[6c] S. Horiuchi , Y. Tokunaga , G. Giovannetti , S. Picozzi , H. Itoh , R. Shimano , R. Kumai , Y. Tokura , Nature 2010, 463, 789–792; 20148035 10.1038/nature08731

[6d] S. Horiuchi , Y. Okimoto , R. Kumai , Y. Tokura , Science 2003, 299, 229–232; 12522245 10.1126/science.1076129

[6e] E. Collet et al., Science 2003, 300, 612–615; 12714737 10.1126/science.1082001

[6f] A. S. Tayi et al., Nature 2012, 488, 485–489. 22914165 10.1038/nature11395

[7] K. Kishikawa , S. Nakahara , Y. Nishikawa , S. Kohmoto , M. Yamamoto , J. Am. Chem. Soc. 2005, 127, 2565–2571. 15725011 10.1021/ja046100c

[8] H. Takezoe , K. Kishikawa , E. Gorecka , J. Mater. Chem. 2006, 16, 2412–2416.

[9] D. Miyajima , F. Araoka , H. Takezoe , J. Kim , K. Kato , M. Takata , T. Aida , Science 2012, 336, 209–213. 22499944 10.1126/science.1217954

[10] C. F. C. Fitié , W. S. C. Roelofs , M. Kemerink , R. P. Sijbesma , J. Am. Chem. Soc. 2010, 132, 6892–6893. 20441217 10.1021/ja101734g

[11] S. Cantekin , T. F. A. de Greef , A. R. A. Palmans , Chem. Soc. Rev. 2012, 41, 6125–6137. 22773107 10.1039/c2cs35156k

[12] H. Zimmermann , R. Poupko , Z. Luz , J. Billard , Z. Naturforsch. 1985, 40, 149–160.

[13] C. Tschierske , Nature 2002, 419, 681–683. 12384683 10.1038/419681a

[14] M. O'Keeffe , S. Andersson , Acta Crystallogr. Sect. A 1977, 33, 914–923.

[15] M. Kristiansen , P. Smith , H. Chanzy , C. Baerlocher , V. Gramlich , L. McCusk , T. Weber , P. Pattison , M. Blomenhofer , H.-W. Schmidt , Cryst. Growth Des. 2009, 9, 2556–2558.

[16] M. Schmidt , J. Wittmann , R. Kress , D. Schneider , S. Steuernagel , H.-W. Schmidt , J. Senker , Cryst. Growth Des. 2012, 12, 2543–2551.

[17] C. Zehe , M. Schmidt , R. Siegel , K. Kreger , V. Daebel , S. Ganzleben , H.-W. Schmidt , J. Senker , CrystEngComm 2014, 16, 9273–9283.

[18] D. A. Keen , A. L. Goodwin , Nature 2015, 521, 303–309. 25993960 10.1038/nature14453

[19] U. Brandt , J. Stolze , Z. Phys. B 1986, 64, 481–490.

[20] E. Gorecka , D. Pociecha , J. Mieczkowski , J. Matraszek , D. Guillon , B. Donnio , J. Am. Chem. Soc. 2004, 126, 15946–15947. 15584712 10.1021/ja044597k

[21] J. Breu , P. Stössel , S. Schrader , A. Starukhin , W. J. Finkenzeller , H. Yersin , Chem. Mater. 2005, 17, 1745–1752.

[22] T. R. Welberry , A. P. Heerdegen , D. C. Goldstone , I. A. Taylor , Acta Crystallogr. Sect. B 2011, 67, 516–524. 22101541 10.1107/S0108768111037542

[23a] A. Sugita , K. Suzuki , S. Tasaka , Chem. Phys. Lett. 2004, 396, 131–135;

[23b] H. Pleiner , H. R. Brand , P. E. Cladis , Mol. Cryst. Liq. Cryst. 2003, 396, 169–176.

[24] A. B. Cairns , M. J. Cliffe , J. A. M. Paddison , D. Daisenberger , M. G. Tucker , F.-X. Coudert , A. L. Goodwin , Nat. Chem. 2016, 8, 442–447. 27102677 10.1038/nchem.2462 PMC4843959

[25] T. Aida , E. W. Meijer , S. I. Stupp , Science 2012, 335, 813–817. 22344437 10.1126/science.1205962 PMC3291483

