# Supplementary material for: Mesoscale Polarization by Geometric Frustration in Columnar Supramolecular Crystals
Source: Angew Chem Int Ed Engl. 2017 Mar 20;56(16):4432–7. doi: 10.1002/anie.201612122 (PMC5574020; doi:10.1002/anie.201612122)
Supplement: Supplementary file 1 — Supplementary [file ANIE-56-4432-s001.pdf]

Angewandte Chemie
Eine Zeitschrift der Gesellschaft Deutscher Chemiker

Supporting Information

Mesoscale Polarization by Geometric Frustration in Columnar Supramolecular Crystals

Christoph S. Zehe, Joshua A. Hill, Nicholas P. Funnell, Klaus Kreger, Kasper P. van der Zwan, Andrew L. Goodwin, Hans-Werner Schmidt,* and Jürgen Senker**

anie_201612122_sm_miscellaneous_information.pdf

Supporting Information

Contents

1 Experimental Details 2
1.1 Synthesis 2
1.2 X-Ray Diffraction Experiments 2
1.3 Quantum Chemical Calculations 3
1.4 Model Generation 3
1.5 Simulation of X-Ray Diffraction Patterns 4
2 Analysis of Single-Crystal X-Ray Diffraction Data 5
2.1 Data Collection and Refinement Details 5
2.2 Discussion of Diffuse Scattering and Twinning 9
3 Estimation of Molecular Dipole Moments 11
4 Comparison of Powder X-Ray Diffraction Data with Simulations Based on Ising Models 12

1 Experimental Details

1.1 Synthesis

All compounds were synthesised according to the procedures reported in the literature.[1] Single crystals of **1**, **2** and **3** were obtained by slow solvent evaporation of a saturated solution using N,N-dimethylformamide under ambient conditions. Single crystals of **4** were grown through sublimation and re-condensation by keeping a small amount (~0.5 g) of sample in a closed glass vessel at 160° C for 3 days.

1.2 X-Ray Diffraction Experiments

Single crystal X-ray diffraction experiments were carried out on a STOE IPDS II instrument (Mo-Kα radiation) equipped with a Ge(111) monochromator under ambient conditions for **2**. The crystal with an approximate size of 0.2 mm × 0.2 mm x 1.5 mm was mounted on a glass tip with glue. Data collection, indexing, space group determination, data reduction and reconstruction of reciprocal space layers were performed with the software package X-Area (Stoe). For **3** and **4**, an Oxford Diffraction (Agilent) Supernova diffractometer (CuKα_1 radiation, graphite monochromator) with an Oxford Cryosystems 700 Plus open-flow nitrogen cryostream[2] at 150 K was used. Single crystals with a size of approximately 0.06 mm × 0.06 mm × 0.63 mm and 0.03 mm × 0.03 mm × 0.28 mm, respectively, were mounted using perfluoropolyether oil. Data collection, indexing, space group determination, data reduction and reconstruction of reciprocal space layers were performed with the software package CrysAlis Pro (Agilent). A face-based analytic absorption correction was applied to the integrated Bragg diffraction intensities.[3]
For structure solution and refinement the software package CRYSTALS[4] was used in all cases. Structure solution using direct methods was performed by the SIR92 package[5] and refinement was done against F^2 as implemented in CRYSTALS. All non-hydrogen atoms were refined with anisotropic displacement parameters, all hydrogen atoms with isotropic displacement parameters. For **2** and **3**, all protons were added geometrically and riding constrains were applied. For **4**, all non-NH hydrogen atoms were added geometrically and riding constraints were applied, while all NH atoms were located by difference Fourier electron density maps and refined using riding constraints as implemented in CRYSTALS. Difference Fourier electron density maps were used to locate split sites and visualised by the MCE interface[6] of CRYSTALS. See section 2.1 for detailed results.
Powder X-ray diffraction measurements were carried out on a STOE STADI P diffractometer equipped with a Ge(111) monochromator using CuKα_1 radiation.

Experiments on powders obtained from finely ground single crystals filled in 0.5 mm capillary tubes were conducted in Debye-Scherrer geometry under ambient conditions.

1.3 Quantum Chemical Calculations

The structures obtained from single crystal refinement were geometry optimised on DFT level with the software package CASTEP[7] using the PBE functional and the Tkatchenko-Scheffler dispersion correction scheme.[8] An electronic cut off energy of 900 eV and a Monkhorst k point grid spacing of 0.07 Å^{-1} was used. Dipole moments were calculated for clusters of molecules extracted from the geometry optimised crystal structures while keeping the geometry fixed with the software package GAUSSIAN09[9] using either the PBE1PBE functional on DFT level and a 6-311++G** basis set and the AM1 method, respectively. The dipole moments $\delta_{AM1}(n_{mol})$ obtained for the semiempirical AM1 method as a function of the stack size n_{mol} were first fitted to those of the PBE functional $\delta_{PBE}(n_{mol})$ for stacks consisting of up to six molecules ($n_{mol} \leq 6$) by least-squares refinement of the coefficients c_1 and c_2 in the equation $c_1 \cdot \delta_{AM1}(n_{mol}) + c_2$. Subsequently, the obtained coefficients were used to correct the AM1 results $\delta_{AM1}(n_{mol})$ of stacks containing more than six molecules ($n_{mol} > 6$).

1.4 Model Generation

Two-dimensional Ising models were generated with a custom Monte Carlo code. The energy was defined by

$$E = J_1 \sum_{\langle i,j \rangle} \sigma_i \sigma_j + J_2 \sum_{\langle\langle i,j \rangle\rangle} \sigma_i \sigma_j \ , \qquad (S1)$$

where single brackets denote a sum over nearest neighbours and double brackets a sum over next nearest neighbours. σ_i = 1 / –1 represent up and down oriented columns. The energy was equilibrated for systems containing 100 x 100 spins with periodic boundary conditions using the Metropolis algorithm[10] with $J_1/kT = 1$, where k denotes the Boltzmann constant and T the simulation temperature. We chose the simplest Ising model being capable of explaining the observed superstructures, which includes two effective coupling constants J_1 and J_2.[11] The entire code is available upon request. For the Ising model for **1**, **3** and **4**, where J_1 and J_2 are equal for all six n.n. and n.n.n., respectively, and where J_1 is positive, only three different ground states exist[11]: i) for $J_2 > 0$ an totally ordered stripe-like pattern; ii) for $J_2 < 0$ a honeycomb phase with only one single domain; iii) for $J_2 = 0$ a degenerated manifold of disordered states result at $T = 0$.

For compound **2**, where the underlying rod packing is pseudo-hexagonal, the coupling constants were allowed to differ in the different crystallographic directions of the two-dimensional lattice (Figure S1c). After testing various combinations, the general requirement of all coupling constants being non-negative was established. To reduce the configuration space the coupling constants J_{01}, J_{11}, J_{10} (interpreted as

J_1 in eqn. S1) and J_{12}, J_{21} and J_{-11} (interpreted as J_2 in eqn. S1), respectively, were restrained to be as similar as possible and the best possible match between simulation and observed diffuse scattering possible was identified (Figure S1c). For these constants, a totally ordered stripe-like pattern results at $T = 0$.

Tab. S1 summarises the energies of the equilibrated models as well as the energies of the corresponding Ising ground states. Since $T \neq 0$, all equilibrium energies are larger than the ground state energies, leading to domains instead of ordered ground state structures.

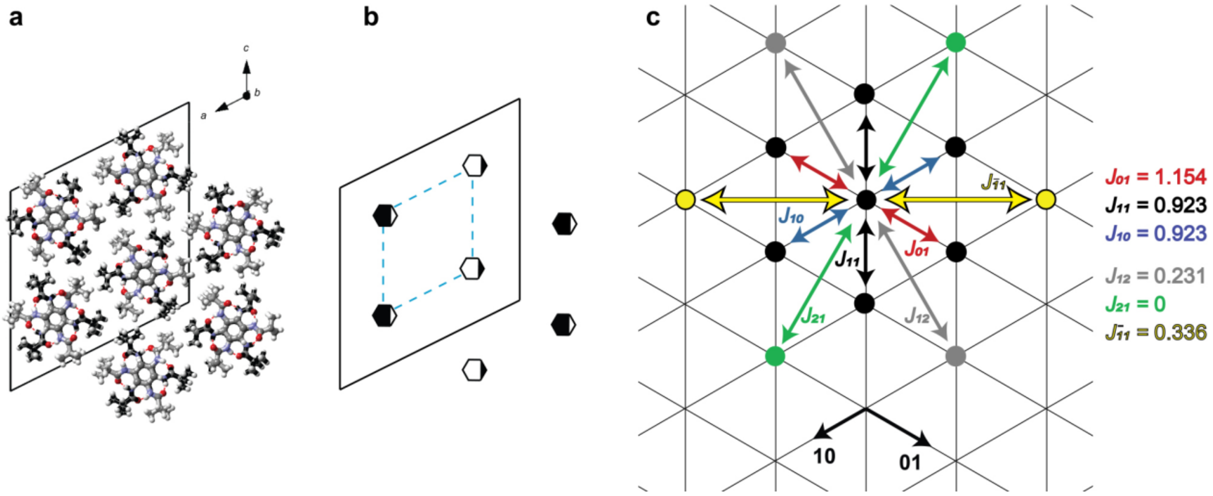

Figure S1: a, The pseudo-hexagonal packing of **2** and **b**, the macrodipole orientations obtained from single-crystal structure solution (cf. Figure 2). **c**, Due to the pseudo-hexagonal rod packing in **2**, an anisotropic Ising model using different coupling constants for different directions is used.

Table S1: Equilibrium energies E_{eq} per atom obtained in the MC simulations of the different Ising models as well as corresponding ground state energies E_{gr} per atom.

Compound	J_1, J_2	E_{eq} / a.u.	E_{gr} / a.u.
**1**	1, 0.1	–0.808	–1.1
**2**	1, 0.2 (average)	–1.035	–1.049
**3**	1, –0.5	–2.130	–4.5
**4**	1, –0.8	–3.387	–5.4

1.5 Simulation of X-Ray Diffraction Patterns

The calculation of powder and single crystal diffraction intensities was facilitated with the programs CrystalDiffract[12] and SingleCrystal,[13] respectively. For all models, the basic shape of diffuse scattering was first inspected using test models being generated by populating the 100 x 100 models with carbon in case of $\sigma_i = 1$ and voids otherwise. The diffuse scattering showed sensitivity towards changes of about 5% of the coupling constants, in particular in the region around $J_{1/2} = 0$.

Subsequently, 36 x 36 subensembles were randomly chosen from the 100 x 100 models and populated with stacks obtained from the single-crystal structure solutions, where every possible subensembles gave the same diffraction pattern. Scattering from these models is identical to that of the test models up to a scale factor. Comparisons of the simulations of powder diffraction patterns using those models with experimental data obtained from ground single crystals are in excellent agreement (see Supplementary Figure S9-S12).

2 Analysis of Single-Crystal X-Ray Diffraction Data

2.1 Data Collection and Refinement Details

Crystallographic information files (.cif) of a) the refinement of the initial structure solution, b) the refinement after adding atoms from difference Fourier electron density maps and c) the result of the geometry optimisation of a) are available in the online version of the paper (Tab. S2) for **2** and **3**. For **4**, the .cif files of the full refinement and of the geometry optimised structure are available.

Key crystallographic details and refinement parameters for the full refinements are given for the compounds **2**-**4** in Tables S3-S5 (for details of data handling and refinement procedures compare section 1.2; for compound **1** see ref. [14]).

For compound **2**, a pseudo-merohedral twin with a twin matrix (0 0 1, 0 -1 0, 1 0 0), swapping the crystallographic *a* and *c* axes, and almost equal twin fractions was detected during refinement. See section 2.2 for a detailed discussion of this twin law and of twinning in general for compounds **2**-**4**.

The diffuse scattering surrounding certain Bragg reflections for **2** and **3** causes errors during integration of the Bragg intensities and leads to large R values, uncommonly large and anisotropic thermal displacement parameters and distorted molecular geometries for these compounds. However, the split sites appear as well-defined residual electron density peaks in Fourier difference maps and the structured diffuse scattering strongly supports our models so that our analyses are meaningful.

Table S2: Summary of the crystallographic information files available in the online version of the paper.

Comp.	File no.	Content	Name of file/data set
**1**		Results of refinement may be obtained from Steurer et al.[14]	-
**1**	1	Geometry optimisation of results from ref [14]	1.cif
**2**	2	Initial refinement	2a.cif
**2**	3	Full refinement including split sites	2b.cif
**2**	4	Geometry optimisation of file no. 2	2c.cif

**3**	5	Initial refinement	3a.cif
**3**	6	Full refinement including split sites	3b.cif
**3**	7	Geometry optimisation of file no. 5	3c.cif
**4**	8	Full refinement	4b.cif
**4**	9	Geometry optimisation of file no. 8	4c.cif

Table S3: Representative single-crystal X-ray diffraction data and refinement results including split sites for **2** (for complete cif file see online version of the paper).

Radiation	Mo-Kα
Formula	C_{21} H_{33} N_3 O_3
M (g/mol)	375.51
Z, calculated density (g/cm^3)	8, 1.060
Crystal Size (mm)	0.2 × 0.2 x 1.5
No. of Reflections	12716
No. of Reflections (I/σ > 2.0)	7826
T / K	293
θ range (°)	1.627-29.358
Completeness (%) / h, k, l range	98.4 / ±38, ±9, ±38
Crystal System	Monoclinic
Space Group	$P2_1/n$
a (Å)	27.948(6)
b (Å)	6.7270(13)
c (Å)	27.953(6)
β (°)	116.43(3)
Goodness of Fit	1.9685
R_p	0.1635
R_p (I/σ > 2.0)	0.1401
wR_p	0.5152
wR_p (I/σ > 2.0)	0.3650
Restraints/parameters	0/ 571
Twin law matrix, twin fractions	(0 0 1, 0 -1 0, 1 0 0), 0.524(4) / 0.476(4)

Table S4: Representative single-crystal X-ray diffraction data and refinement results including split sites for **3** (for complete cif file see online version of the paper).

Radiation	Cu-Kα
Formula	$C_{33} H_{57} N_3 O_3$
M (g/mol)	543.83
Z, calculated density (g/cm^3)	6, 1.055
Crystal Size (mm)	0.061 × 0.061 × 0.634
No. of Reflections	7099
No. of Reflections (I/σ > 2.0)	5664
T / K	150
θ range (°)	3.466-76.126
Completeness (%) / h, k, l range	99.4 / -31/+36, -36/+37, ±8
Crystal System	Hexagonal
Space Group	$P6_3$
a (Å)	29.4501(9)
c (Å)	6.8393(2)
Goodness of Fit	1.1565
R_p	0.1175
R_p (I/σ > 2.0)	0.1026
wR_p	0.2751
wR_p(I/σ > 2.0)	0.2574
Restraints/parameters	0 / 705

Table S5: Representative single-crystal X-ray diffraction data and refinement results including split sites for **4** (for complete cif file see online version of the paper).

Radiation	Cu-Kα
Formula	$C_{18} H_{24} F_3 N_3 O_3$
M (g/mol)	387.4
Z, calculated density (g/cm^3)	6, 1.242
Crystal Size (mm)	0.026 × 0.026 × 0.284
No. of Reflections	2278
No. of Reflections ($I/\sigma > 2.0$)	2171
T / K	150
θ range (°)	3.802-51.535
Completeness (%) / h, k, l range	99.9 / ±23, ±23, ±6
Crystal System	Hexagonal
Space Group	$P6_3$
a (Å)	23.2502(4)
c (Å)	6.63730(10)
Goodness of Fit	1.0069
R_p	0.0324
R_p ($I/\sigma > 2.0$)	0.0305
wR_p	0.0798
wR_p($I/\sigma > 2.0$)	0.0780
Restraints/parameters	9 (N-H hydrogen restraints) / 253

2.2 Discussion of Diffuse Scattering and Twinning

First, we note that the diffuse scattering for compounds **2** and **3** is confined to reciprocal lattice planes perpendicular to the stacking direction (Figure S2; **4** does not exhibit any diffuse scattering). This implies that the columns are not disordered along the stacking direction within the coherence length of the beam. Hence, only macroscopic twinning may occur along the column axis, which cannot be the origin of split positions. We will discuss the possible twin operations for compounds **2**-**4** in the following and show that the only possible origin of the split positions and the diffuse scattering is microscopic disorder.

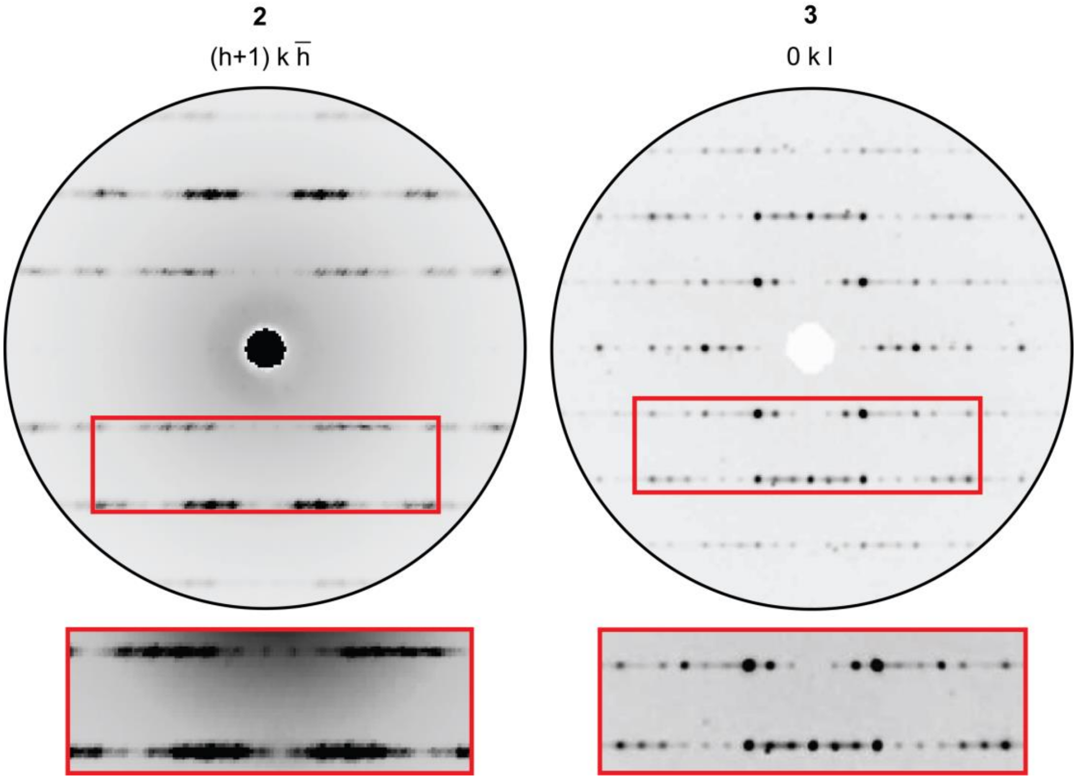

Figure S2: Planes of the reciprocal space reconstruction perpendicular to those presented in Figure 3 of the main paper for compounds **2** and **3**. The red boxes show magnifications of selected regions.

For compounds **3** and **4**, the non-centrosymmetric space group $P6_3$ was determined. In the case of twinning by merohedry, the two twin components are related by a symmetry operation, which belongs to the point group of the lattice (holohedry) but not to the point group of the crystal. This may be separated in class I and class II merohedral twinning. In class I twins by merohedry (inversion twins), the twin symmetry operation is part of the Laue group 6/m but not of the point group 6 of the crystal. This means that the m plane of the Laue group (or, equivalently, the inversion centre) is the twin symmetry operation. Since the diffraction patterns exhibit intrinsic inversion symmetry (i.e. Friedel pairs are equivalent since anomalous scattering was not detectable / refinement of Flack parameter was indifferent since heavy atoms are absent) this kind of twinning does not affect the observed diffraction intensities. It

thus cannot be the cause of the split positions for **3.** Moreover, since such a twinning would require a break in the hydrogen bond network of each stack, it seems energetically unfavourable.

In case of class II twinning by merohedry, the twin symmetry operation is not part of the Laue group but part of the holohedry. In this case, the diffraction intensities are affected by exact coincidence of non-identical diffraction patterns. Such a twinning, however, cannot lead to an apparent space group $P6_3$ (see ref. [15]) and hence it can be excluded for **3** and **4** (moreover, such a twinning would also retain the macrodipole directions and may not cause the well-defined split positions).

For compound **2**, the centrosymmetric space group $P2_1/n$ was determined. For this space group, twinning by merohedry is not possible. However, since the *a* and *c* axis are basically identical in length, the lattice exhibits approximately a higher point group symmetry of 2/*m* 2/*m* 2/*m* (orthorhombic) compared to the point group symmetry 2/*m* of the space group $P2_1/n$ and hence twinning by pseudomerohedry is likely. Indeed, it was found during refinement that consideration of a twin matrix (0 0 1, 0 -1 0, 1 0 0), swapping the crystallographic *a* and *c* axes (Figure S3a,b), leads to a significant drop of more than 10 % of the crystallographic *R* value. This, however, means that the twinning occurs on macroscopic length scales, which does not influence the shape of the diffuse scattering, since it is mapped on itself (Figure S3c).

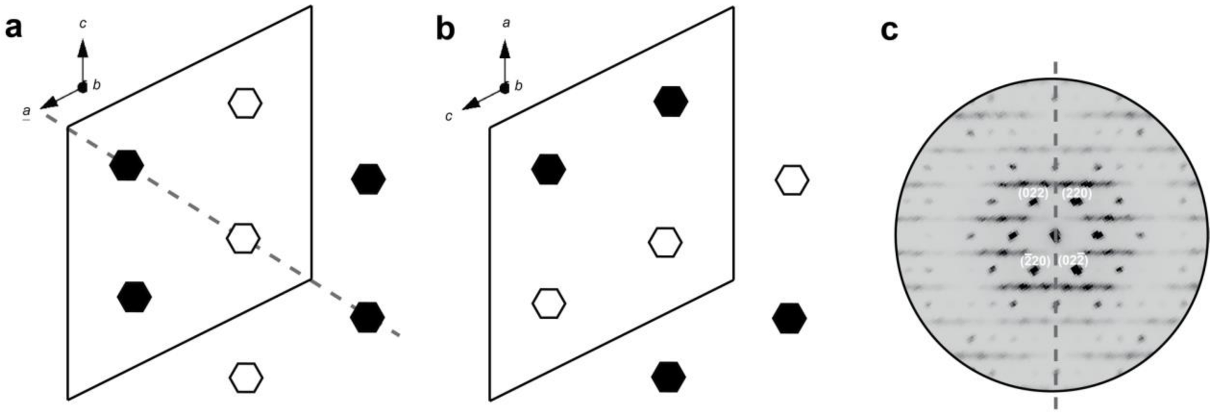

Figure S3: a, The twin operation (0 0 1, 0 -1 0, 1 0 0), swapping the crystallographic *a* and *c* axes in the structure of **2**, means a reflection of the structure on a mirror plane containing the *b* axis and running through the corners of the unit cell (grey dashed line). **b**, The twin component produces by this twinning operation shows the same stripe-like macrodipole pattern, but with a different orientation. The minor parts of the split positions are omitted for clarity. **c**, The diffuse scattering observed maps onto itself by this twinning operation.

Twinning by pseudo-merohedry through a 120° rotation about the *b* axis was not detected during refinement; in addition, such a twinning would not map the diffuse scattering onto itself but create a checked pattern, which can obviously be excluded.

As a consequence, the only possible origin of the split sites for **2** and **3** is macrodipole disorder on a length scale similar to that of the coherence length of the beam.

3 Estimation of Molecular Dipole Moments

We estimate the average molecular dipole moments of an infinite stack for compounds **1**, **2** and **4** by quantum chemical calculations. First, the crystal structures obtained from structure solutions (where only the major part of the split positions were taken into account) were geometry optimised by DFT methods using the PBE functional to correct the distorted molecular geometries caused by the diffuse scattering during refinement. Next, accurate dipole moments were calculated by DFT methods using the PBE1PBE functional for stacks containing up to six molecules (Figure S4a), which constitutes the computational limit. The average dipole moment per molecule increases steadily with increasing number of molecules per stack (n_{mol}) due to cooperative effects.[16] Convergence can be reached only using semiempirical AM1 calculations for up to twenty molecules; however, the magnitude of the dipole moments differs markedly for both techniques. Therefore, we fitted the less precise AM1 results $\delta_{AM1}(n_{mol})$ to the more accurate PBE1PBE calculations $\delta_{PBE}(n_{mol})$ (Figure S4a) by refining a the coefficients c_1 and c_2 of equation $\delta_{PBE}(n_{mol}) = c_1 \cdot \delta_{AM1}(n_{mol}) + c_2$. Subsequently, the obtained coefficients were used to correct the AM1 results $\delta_{AM1}(n_{mol})$ of stacks containing more than six molecules ($n_{mol} > 6$). By this, we can estimate the average molecular contribution to the macrodipole to 12.2 D, 10.8 D and 6.6 D for **1**, **2** and **4**, respectively.

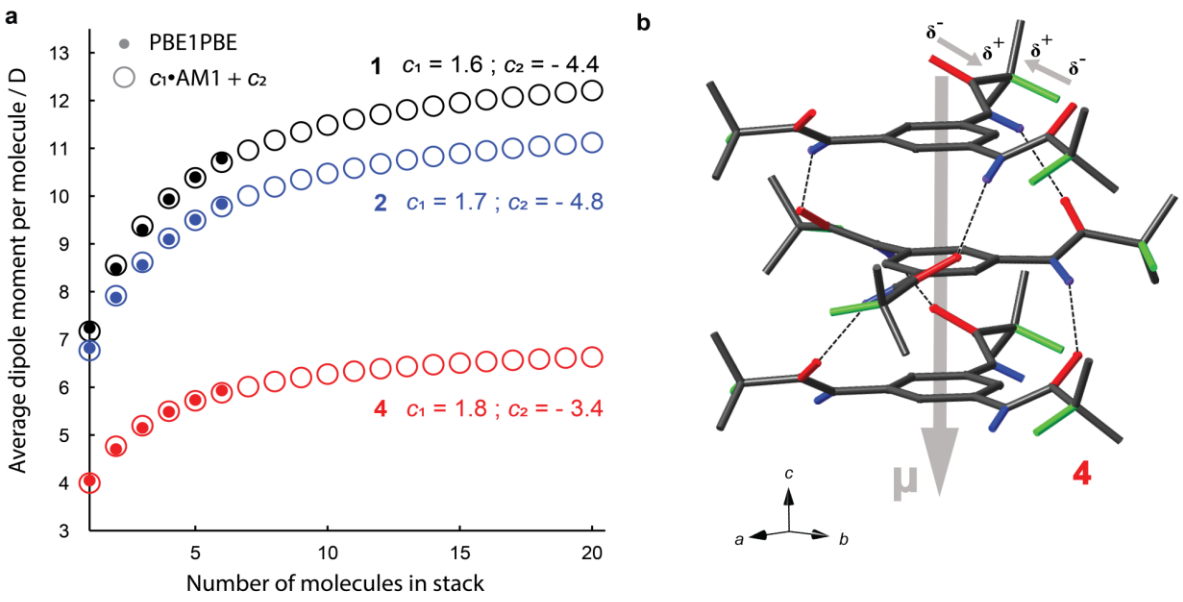

Figure S4. a, Average dipole moment per molecule for stacks of varying sizes isolated from the crystal structure without geometry optimisation. The filled circles correspond to calculations $\delta_{PBE}(n_{mol})$ using the PBE1PBE functional and are limited to six molecules due to calculation time. The open circles are values $\delta_{AM1}(n_{mol})$ obtained by the semiempirical AM1 method and are scaled by the equation $\delta_{PBE}(n_{mol}) = c_1 \cdot$

$\delta_{AM1}(n_{mol})$ $+c_2$ (open circles), where the displayed c_1 and c_2 values are constants for each compound and n_{mol} demarks the number of molecules in the stack. **b**, Detail of the crystal structure of **4** where the N-H, C-F and C=O bonds are blue, green and red, respectively. The polarities of the C=O and C-F bonds are indicated. All non-NH protons are omitted for clarity.

4 Comparison of Powder X-Ray Diffraction Data with Simulations Based on Ising Models

Figures S9-S12 show experimental powder X-ray diffraction data from ground single-crystals in comparison to simulated powder patterns based on the same models used for simulation of the single-crystal X-ray diffraction patterns (Figure 3a). They indicate that the basic underlying structure, the (pseudo-)hexagonal rod packing, is correct for all crystals of the samples and not only for the selected ones chosen for single-crystal diffraction experiments.

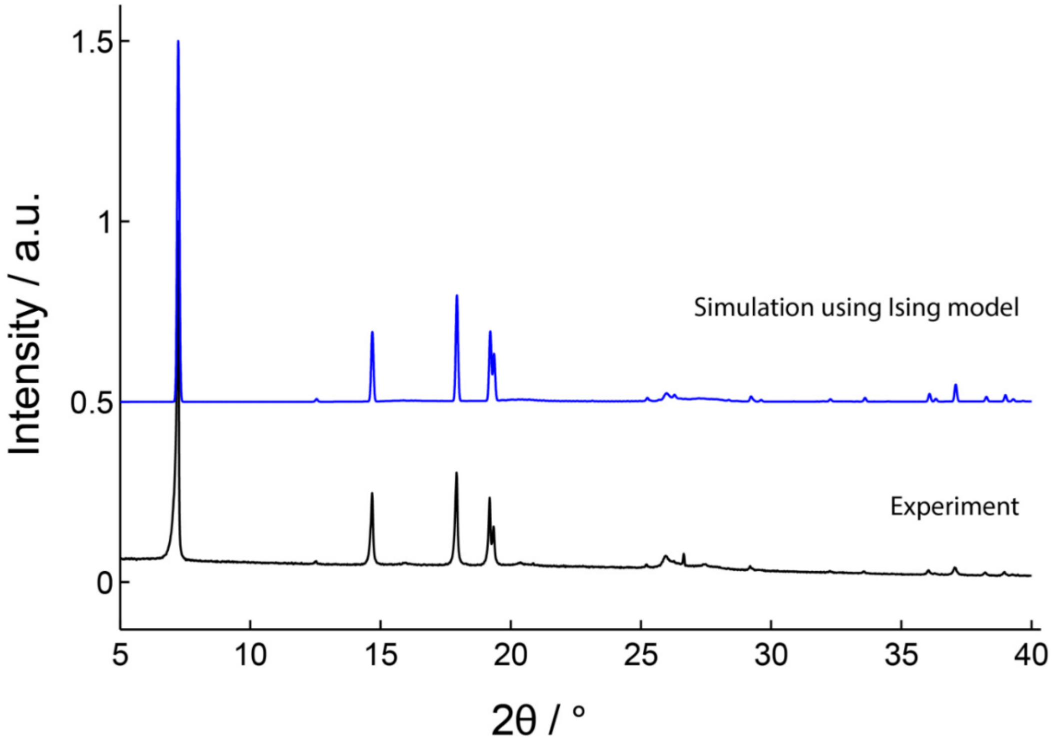

Figure S5: Comparison of powder X-ray diffraction experiment (black, bottom) of **1** with simulations of powder X-ray diffraction patterns based on the Ising models for **1** (blue, top).

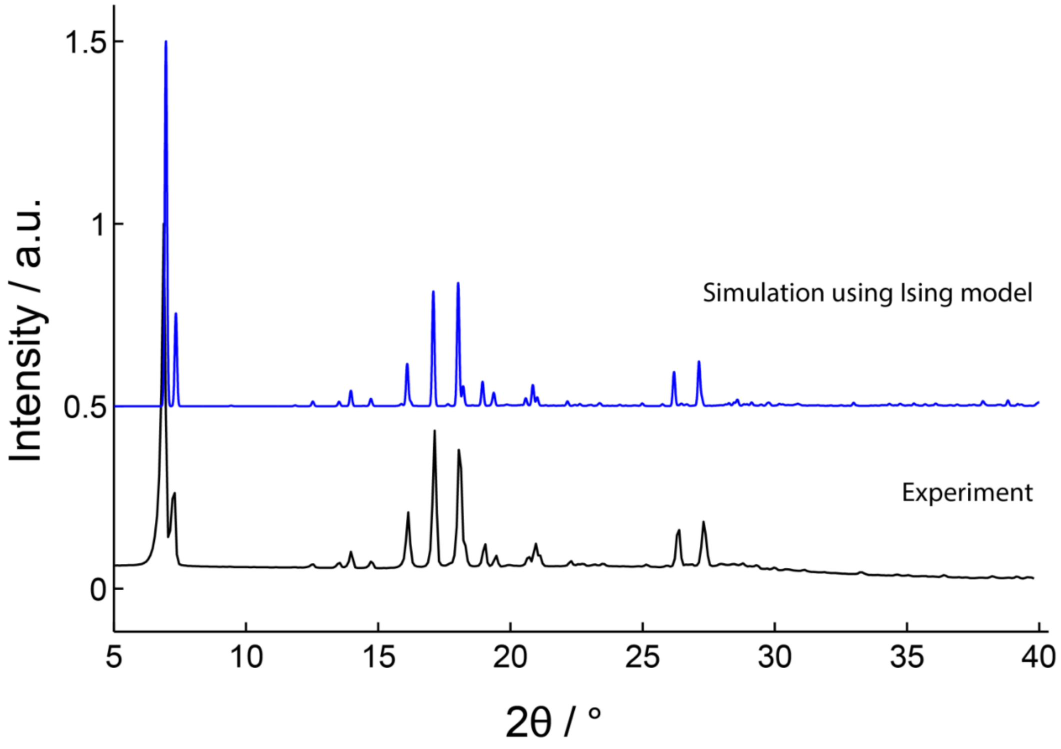

Figure S6: Comparison of powder X-ray diffraction experiment (black, bottom) of **2** with simulations of powder X-ray diffraction patterns based on the Ising models for **2** (blue, top).

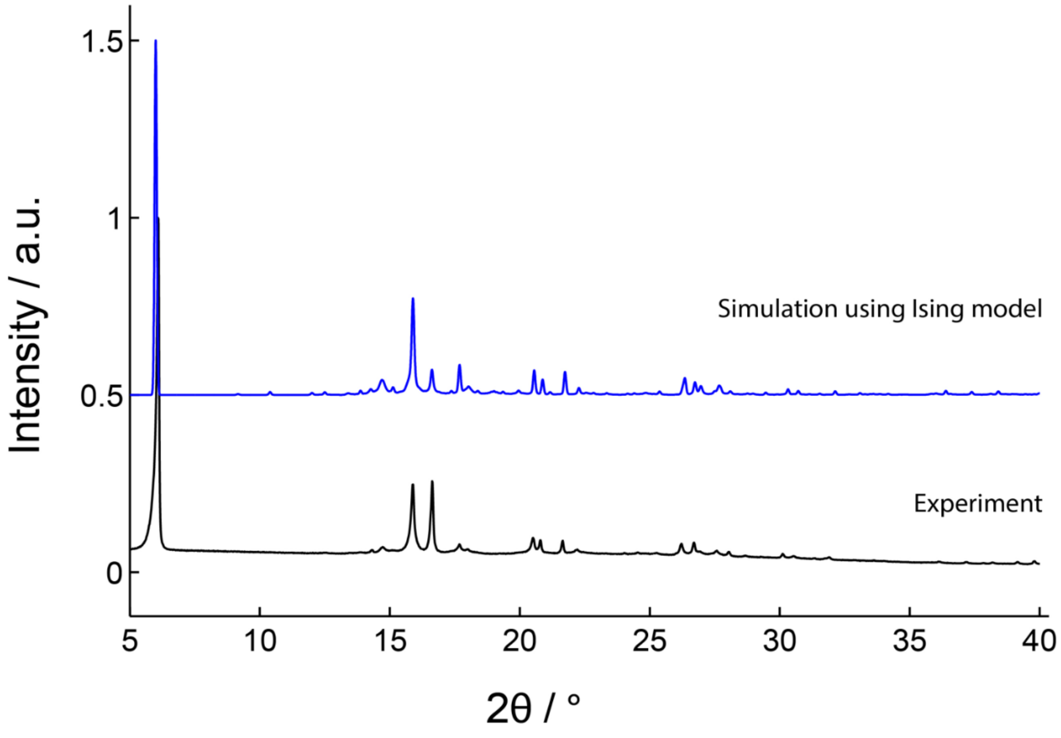

Figure S7: Comparison of powder X-ray diffraction experiment (black, bottom) of **3** with simulations of powder X-ray diffraction patterns based on the Ising models for **3** (blue, top).

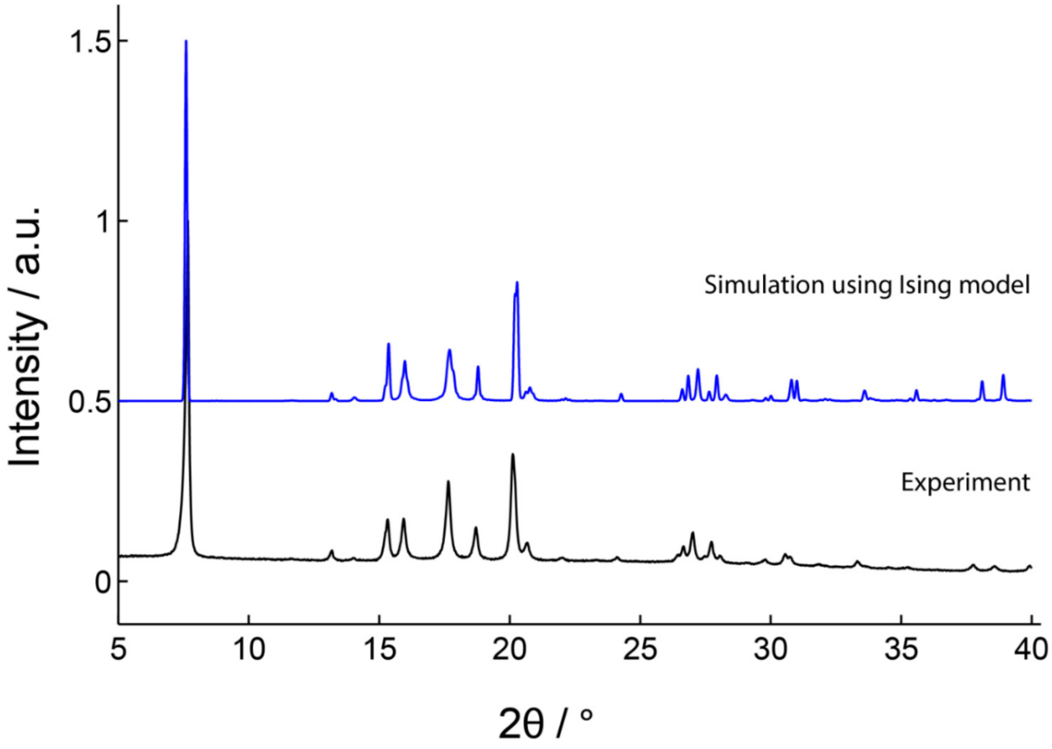

Figure S8: Comparison of powder X-ray diffraction experiment (black, bottom) of **4** with simulations of powder X-ray diffraction patterns based on the Ising models for **4** (blue, top).

References

[1] a) C. Zehe, M. Schmidt, R. Siegel, K. Kreger, V. Daebel, S. Ganzleben, H.-W. Schmidt, J. Senker, *CrystEngComm* **2014**, *16*, 9273–9283; b) F. Abraham, S. Ganzleben, D. Hanft, P. Smith, H.-W. Schmidt, *Macromol. Chem. Phys.* **2010**, *211*, 171–181.

[2] J. Cosier, A. M. Glazer, *J. Appl. Crystallogr.* **1986**, *19*, 105–107.

[3] R. C. Clark, J. S. Reid, *Acta Crystallogr. A* **1995**, *51*, 887–897.

[4] P. W. Betteridge, J. R. Carruthers, R. I. Cooper, K. Prout, D. J. Watkin, *J. Appl. Crystallogr.* **2003**, *36*, 1487.

[5] A. Altomare, G. Cascarano, C. Giacovazzo, A. Guagliardi, M. C. Burla, G. Polidori, M. Camalli, *J. Appl. Crystallogr.* **1994**, *27*, 435.

[6] J. Rohlíček, M. Hušák, *J. Appl. Crystallogr.* **2007**, *40*, 600–601.

[7] S. J. Clark, M. D. Segall, C. J. Pickard, P. J. Hasnip, M. I. J. Probert, K. Refson, M. C. Payne, *Z. Kristallogr.* **2005**, *220*, 567–570.

[8] E. R. McNellis, J. Meyer, K. Reuter, *Phys. Rev. B* **2009**, *80*, 205414.

[9] M. J. Frisch, G. W. Trucks, H. B. Schlegel, G. E. Scuseria, M. A. Robb, J. R. Cheeseman, G. Scalmani, V. Barone, B. Mennucci, G. A. Petersson et al., *Gaussian09; Revision C.01*, Gaussian Inc., Wallingford, Conneticut, US, **2010**.

[10] N. Metropolis, A. W. Rosenbluth, M. N. Rosenbluth, A. H. Teller, E. Teller, *J. Chem. Phys.* **1953**, *21*, 1087–1092.

[11] U. Brandt, J. Stolze, *Z. Phys. B Con. Mat.* **1986**, *64*, 481–490.

[12] D. C. Palmer, *CrystalDiffract 6*, CrystalMaker Software Ltd, Begbroke, Oxfordshire, England, **2013**.

[13] D. C. Palmer, *SingleCrystal 2*, CrystalMaker Software Ltd, Begbroke, Oxfordshire, England, **2010**.

[14] A. Simonov, T. Weber, W. Steurer, *J. Appl. Crystallogr.* **2014**, *47*, 2011–2018.

[15] C. Giacovazzo, *Fundamentals of crystallography*, Oxford University Press, Oxford, New York, **2011**.

[16] I. A. W. Filot, A. R. A. Palmans, P. A. J. Hilbers, R. A. van Santen, E. A. Pidko, T. F. A. de Greef, *J. Phys. Chem. B* **2010**, *114*, 13667–13674.
